# Emotional eating: elusive or evident? Integrating laboratory, psychometric and daily life measures

**DOI:** 10.1007/s40519-023-01606-8

**Published:** 2023-09-13

**Authors:** Rebekka Schnepper, Jens Blechert, Ann-Kathrin Arend, Takuya Yanagida, Julia Reichenberger

**Affiliations:** 1 Faculty of Psychology, Department of Health Psychology and Centre for Cognitive Neuroscience, Paris-Lodron-University of Salzburg, Hellbrunner Str. 34, 5020 Salzburg, Austria; 2 Department of Psychosomatics, University Hospital Basel, Hebelstr. 2, 4056 Basel, Switzerland; 3 Faculty of Psychology, Department of Developmental and Educational Psychology, University of Vienna, Universitaetsstr. 7, 1010 Vienna, Austria

**Keywords:** Emotional eating, Restrained eating, Eating disorders, Confirmatory factor analysis, Measure coherence

## Abstract

**Purpose:**

Emotional eating (EE) refers to eating in response to (negative) emotions. Evidence for the validity of EE is mixed: some meta-analyses find EE only in eating disordered patients, others only in restrained eaters, which suggest that only certain subgroups show EE. Furthermore, EE measures from lab-based assessments, ecological momentary assessment (EMA), and psychometric measures often diverge. This paper tested whether the covariance of these three different EE methods can be modeled through a single latent variable (factorial validity), and if so, how this variable would relate to restrained eating (construct validity), Body-Mass-Index (BMI), and subclinical eating disorder symptomatology (concurrent validity).

**Methods:**

102 non-eating disordered female participants with a wide BMI range completed EE measures from three methods: psychometric questionnaires, a laboratory experiment (craving ratings of food images in induced neutral vs. negative emotion) and EMA questionnaires (within-participant correlations of momentary negative emotions and momentary food cravings across 9 days). Two measures for each method were extracted and submitted to confirmatory factor analysis.

**Results:**

A one-factor model provided a good fit. The resulting EE_lat_ factor correlated positively with subclinical eating disorder symptoms and BMI but not with restrained eating.

**Conclusions:**

The one-factor solution shows that the EE construct can validly be assessed with three different methods. Individual differences in EE are supported by the data and are related to eating and weight problem symptomatology but not to restrained eating. This supports learning accounts of EE and underscores the relevance of the EE construct to physical and mental health.

**Level of evidence:**

II (Evidence obtained from well-designed controlled trials without randomization).

**Supplementary Information:**

The online version contains supplementary material available at 10.1007/s40519-023-01606-8.

## Introduction

Eating more in response to negative emotions may overwrite homeostatic body signals (i.e., hunger and saturation) that normally regulate food intake. This process of emotional eating (EE) is reinforced by the experience of reward from consuming foods high in sugar, salt, and/or fat, therefore, potentially regulating stress and negative emotions [1, 2]. Clinical significance of EE is supported by its relationship with the development and maintenance of eating disorders (e.g., [3]), possibly because negative emotions might trigger binge eating in patients with bulimia nervosa or binge-eating disorder and extreme dieting in anorexia nervosa (AN; [4–6]). EE is further positively related to Body-Mass-Index (BMI), pointing to its relevance with weight disorders [7, 8].

EE has triggered considerable interest and empirical investigations since its inception more than 70 years ago [9]. Different, albeit related theories on the nature of EE have been proposed. Physiological EE theories suggest various relationships of a stress-related activation of the hypothalamic pituitary adrenal (HPA) axis and glucocorticoids or reward pathways leading to overeating but also undereating [10]. Chronic stress and negative emotions might lead to a vicious cycle of overeating in response to this and then experiencing more stress and negative emotions due to negative health consequences of an unhealthy diet [11]. Furthermore, there are several psychological EE theories. Learning theories postulate that EE represents a dysfunctional emotion regulation strategy, where negative emotions are compensated through the positive reward obtained from tasty foods instead of utilization of more adaptive strategies [12]. Such preferences in regulation strategy are thought to result from (early) learning experiences [13]. In contrast, cognitive theories attribute EE to a disinhibition of dietary restraint. Accordingly, rigid weight loss dieting strategies impose strict boundaries on eating and ban certain high-calorie foods; a process that consumes self-regulatory resources. As a result, when stress or negative emotions compete for these self-regulatory resources, dieters temporarily abandon these limits in an all-or-nothing fashion and overeat [14, 15], only to return to the strict boundaries thereafter, closing a vicious cycle [16]. All of these theories have substantial overlap, so that it seems difficult to disentangle them without acknowledging further aspects, such as simultaneous cognitions, post-eating emotions and inter-individual differences.

On top of these different perspectives on EE with some overlaps, but also disagreements, the empirical basis for the EE concept has been heterogeneous since the first studies in this research field in the 1950s. Some researchers have doubted whether EE exists at all, and if it does, whether actual food intake increases or decreases in response to negative emotions and how this can be measured [17]. Reviewing the EE literature, the authors found broad variability and inconsistency regarding EE which might be due to methodological variability (e.g., emotion induction method, food availability) but also the validity of the EE construct. Indeed, several studies found no connection between self-reported EE and increased food intake in negative mood, neither in healthy-weight [18] nor in overweight populations [19]. One proposed reason is that in negative mood, one might be more concerned about one's own eating behavior and thus more aware of overeating, which might not be noticed in a more positive mood [20, 21]. Furthermore, overeating might not be specific to negative emotions, but rather generalize to any situation that draws (non-emotional) attention to food. This non-emotional overeating is then attributed to stress and negative emotions in retrospect, so that EE serves as a license, explanation or excuse for overeating [22]. Hence, a review concluded that self-reported EE does not translate to actual increased food intake in response to negative emotions [17].

These conceptual and empirical disagreements in the EE literature highlight the need to better understand whether normal-weight individuals without an eating disorder show measurable EE with a clear cause–effect relation. Thus, the first aim of this paper was to investigate the factorial validity of EE and whether different measures from complementary methods (‘multi-measure’ approach) would cohere on a unidimensional latent EE factor. Using data from this multi-measure EE, we also contrast learning theories against cognitive theories (i.e., restrained eating). A meta-analysis stated that self-reported EE did not predict actual overeating in laboratory studies, while restrained eating did so [16]. Evers et al. [16] concluded that EE might be a consequence of disinhibited restraint and that disinhibited restraint might also be related to weight problems. Restraint can have different degrees of severity and can become the core of eating disorder symptomatology, such as in AN. Hence, the second aim of this paper was to examine the construct validity of our EE factor by analyzing whether subclinical levels of restrained eating might also covary with our multi-measure EE.

The third aim of this paper was to investigate the concurrent validity of EE, i.e., how our multi-measure EE relates to BMI and subclinical eating disorder symptomatology. Evidence on the relationship between EE and weight problems is mixed: a study by Brogan and Hevey [19] found no correlation between a higher BMI and EE, while other studies reported a correlation between higher BMI and EE [7]. A review concluded that higher EE and higher BMI might be positively correlated and that targeting EE in interventions might facilitate weight loss [23]. However, the authors also criticize that research on the relationship between EE and weight is still at an early stage. Thus, we included participants with a wide range in BMI. As mentioned above, EE might be related to the development and maintenance of eating disorders. Thus, we included a measure for eating symptomatology to test whether our multi-measure EE would relate to subclinical levels of this symptomatology.

Due to the conflicting findings of previous studies on the validity of EE, Evers et al. [16] recommend utilization of various methods of EE, e.g., by combining self-reported EE with behavioral measures, as well as laboratory with longitudinal daily life measures. The present paper follows this advice. Specifically, we used ecological momentary assessment (EMA) in daily life, self-report measures (questionnaires), and emotion-induced food-cue reactivity in an experimental setting. Daily life measures have the advantage of measuring eating behavior in a naturalistic environment, and limiting recall biases as behaviors and experiences are measured close to their occurrence. Yet, EMA does not involve manipulation of emotion and thus cannot exclude third variables to ascertain causality. Psychometric measures are popular due to their economy and their direct relationship with individuals’ self-concept, but they could be affected by self-report biases. Only laboratory-based emotion induction studies can ensure causality of effects of emotions on eating.

Thus, this paper aims at a comprehensive understanding of EE by investigating whether these different, but potentially complementary assessment methods provide convergent evidence for EE. First, to obtain a measure-independent assessment of EE we combined data from questionnaires, emotion-modulated food-cue reactivity in the laboratory, and EMA by means of confirmatory factor analysis (CFA). A one-factor solution would support factorial validity of the EE construct. In a second step, we examined the construct validity of our ‘multi-measure’ EE: as a test of cognitive theories of EE, the latent EE factor (EE_lat_) was correlated with a questionnaire measure of restrained eating, addressing findings that EE is most prominently seen in restrained eaters. Third, we tested concurrent validity by relating EE_lat_ to BMI and subclinical eating disorder symptomatology. Thus, our sampling strategy overrepresented individuals with higher BMIs but excluded individuals with eating disorders. In short, these three research questions ask about EE ‘do different EE measures cohere?’ and ‘what are its correlates?’.

## Methods

### Participants

Participants were recruited for a study at the University of Salzburg, Austria which investigated the influence of emotions on eating behavior via newspaper articles, university newsletters, web portals, flyers, and word of mouth. Data were collected between October 2016 and July 2019. Recruitment aimed for a wide range on BMI, particularly towards a higher BMI, so that recruitment material and outlet was adopted accordingly (e.g., recruiting at obesity self-help forums). They were required to be female, as women have been reported to be at a higher risk to develop an eating disorder [24], to be more likely to experience potentially pathological food craving [25], and to overeat in response to stress or negative emotions [15, 26]. Exclusion criteria were not eating meat (as some of the foods presented contained meat), having food allergies, diabetes and other disorders affecting digestion and diet habits, a current/lifetime eating disorder, current substance abuse, and neurological disorders. From *N* = 103 participants (with experimental, EMA and questionnaire data), one subjects was excluded for completing less than 50% of the EMA signals. The final sample consisted of *N* = 102 participants with a mean compliance of 86% (SD = 10.6%, see Table 1 for further sample characteristics). This sample size was determined by sample size calculations of other studies [6, 7, 27–34]. For both the EMA and the laboratory part of the study, participants were given study credits, 30–45€ (depending on EMA compliance), or 30€ and an individualized feedback on their EMA data (e.g., including figures about their individual emotion and eating behavior courses and stressor types, psychoeducation about a healthy diet and smaller interventions about recognizing stress responses or ‘urge surfing’) as compensation.

**Table 1:** Characteristics of the final sample (*N* = 102)

Variable	*M* (*SD*)
Age (in years)	24.8 (6.9)
Body mass index (BMI; in kg/m^2^)	25.9 (7.1)
Years of education	14.6 (2.8)

Variable	*N* (%)
Underweight (BMI < 18.5)	5 (4.9)
Normal weight (BMI 18.5–25)	57 (55.9)
Overweight (BMI > 25)	19 (18.6)
Obese (BMI > 30)	21 (20.6)
Highest degree	
High school	65 (63.7)
College	22 (21.6)
Other	15 (14.7)

Values show means (*M*) and standard deviations (SD) or number of individuals (*N*) and percentage (%)

### Procedure

After being screened for inclusion and exclusion criteria, participants completed a battery of online questionnaires on eating styles and pathologies, emotion regulation, mood, and demographics (Data previously used in [7, 29]). Subsequently, participants were guided through the installation and usage of a smartphone app and were provided a manual that informed about the content and handling of the smartphone app. After installing the app, the 10 day EMA phase started (data previously reported in [35]). The first day served as a “practice day” to become familiar with the app, with data not being analyzed. Throughout the EMA phase, compliance was being monitored by staff. Participants were sent six equidistant prompts at 9 a.m., 11.30 a.m. 2 p.m., 4.30 p.m., 7 p.m., and 9.30 p.m. The last prompt contained additional end-of-the-day questions.

At the end of this period, participants were asked to take part in a laboratory experiment, which took place at 3 pm after participants had a standardized lunch at home (5 different options, hot or cold, sweet or savory, convenience of fresh cooked, mostly vegetarian, easily available for the sample, each ~ 550 kcal) to minimize variance in circadian rhythm and hunger. At the beginning of the experiment, participants gave informed consent and completed baseline measures (for *n* = 3 underage participants, parents’ consent was obtained). Next, the experimenter attached physiological sensors, participants rated their current hunger, and completed an interoception task (∼10 min; data previously reported in [30]). Then, the EE task was conducted. After the EE task (described in detail below; data previously reported in [6, 28, 31]), a decision-making task (data previously reported in [32–34]) and a taste test followed.

### Measures

*Dutch eating behavior questionnaire [DEBQ German (FEV-I); 33 Items *[36]. The DEBQ measures eating behavior on three subscales: emotional eating, restrained eating, and external eating. The current study used mean scores of the 13-item emotional eating subscale (DEBQ_emo_) and of the 10-item restrained eating subscale. Items are answered on a 5-point Likert scale ranging from 1 = “never” to 5 = “very often”. An example item for the emotional eating subscale would be “Do you have the desire to eat when you are irritated?”; an example item for the restrained eating subscale would be “Do you try to eat less than you would like to eat at mealtimes?”. Cronbach’s α was 0.88 for the emotional eating subscale and 0.88 for the restrained eating subscale.

*Salzburg emotional eating scale (SEES) *[8]. The SEES measures changes in the amount of food intake in response to specific emotions (happiness, anxiety, anger and sadness) with 20 items. The negative subscales anxiety, anger, and sadness were averaged to a ‘negative emotional eating’ scale, or SSES_neg_, which has demonstrated high reliability in a previous study [6] and in the current data (Cronbach’s α = 0.85). Items (e.g., “When I am upset, …") are answered on a 5-point Likert scale from 1 = “I eat much less than usual” to 5 = “I eat much more than usual”.

*Eating disorder examination-questionnaire 8 (EDE-Q8) *[37]. The EDE-Q8 is a shortened 8-item form of the original questionnaire which assesses global eating disorder symptomatology, e.g., by inquiring about food preoccupation, body dissatisfaction, dieting attempts, and guilt from eating. Items are answered on a 7-point scale (either indicating the number of days within the past 4 weeks or the strength of a feeling towards the own body). Items are averaged to one overall score, with higher scores indicating higher eating disorder symptomatology. Cronbach’s α was 0.89 for the EDE-Q8.

*BMI* Participants either provided their weight and height (*n* = 61) or were weighed and measured when arriving for the laboratory experiment (*n* = 41). With this information, their BMI was calculated.

*EMA—end-of-the-day emotional eating and current craving and negative affect.* During the EMA phase, participants stated their current stress level, negative affect, food craving, and eating episodes six times per day. In this study, only negative affect and food craving were included, which were answered on a visual analogue scale ranging from 0 = “not at all” to 100 = “very much”. For this study, an overall score of the six items assessing negative affect was computed. Participants were asked whether right now they felt irritated, bored, worried, depressed, dissatisfied with oneself, and nervous/stressed. To assess food craving, participants were asked “Do you have a desire to eat something tasty right now?”.

The last prompt at the end of each day contained additional questions on eating behavior, stress, physical activity, and mood. One question asked “Did your mood have an influence on how much you ate today?”, which can be seen as an end-of-day review of daily emotional eating. This question was answered on a visual analogue scale ranging from 0 = “I ate less” to 100 = “I ate more” (with “no influence” as a neutral midpoint).

*Laboratory measures of emotional food cue reactivity—desire to eat and pleasantness.* For the EE task, participants were interviewed about a recent situation that elicited negative emotions, such as sadness or frustration (excluding traumatic events). This situation was then used by the experimenter to generate a script of eight sentences, which were presented to the participant during the EE task to induce negative emotions. Furthermore, participants chose one out of two neutral scripts (eight sentences about either brushing their teeth or going to work/school). In both conditions of the EE task (neutral, negative; alternated in order across participants), scripts were first read out to the participant by the experimenter via speakers. Then, the computer task started, in which sentences were presented on screen in-between the presentation and rating of food and object pictures (26 pictures each). Within each condition, the scripts and all pictures were repeated once out of which one was rated. For all pictures, participants rated *pleasantness* on a visual analog scale ranging from 0 = “very unpleasant” to 100 = “very pleasant”. Food pictures were additionally rated for current *desire to eat* on a visual analog scale ranging from 0 = “no desire to eat” to 100 = “strong desire to eat”. Parallel to the EMA question inquiring about tasty food, only the ratings of the 13 high calorie foods were included in the analyzes (see Online Appendix 1 for pictures and numbers of the 13 foods from the foodpics_data set [38]).

To check whether the laboratory experiment induced negative emotions, we examined emotionality before and after the conditions (i.e., before, after condition 1, after condition 2) with the Positive and Negative Affect Schedule [39]. Similar to previous results of the same data set, results showed a successful emotion induction: mean values of the subscale for negative emotions were highest after the negative condition (*M* = 16; *SD* = 5.92) and differed significantly from values after baseline (*M* = 13.6; *SD* = 4.69) and after the neutral condition (*M* = 12.1; *SD* = 3.22) at *p* < 0.001.

### Statistical analysis

From each method (i.e., questionnaire, laboratory, EMA), we obtained two measures as follows. First, for the method questionnaires we obtained the measures DEBQ_emo_ and SEES_neg_. Second, for the method laboratory we derived two rating difference scores: for pleasantness, the ratings for food pictures in the neutral condition were subtracted from those in the negative condition (additionally, the respective ratings object pictures were subtracted, i.e., a double difference score). This ‘pleasantness_hcal_’ measure indexes the emotion-related change in food specific pleasantness. Similarly, desire to eat ratings the neutral condition were subtracted from those in the negative condition, and included as ‘DTE_hcal_’ (no DTE available for objects (see [28]). Third, for the method EMA we derived two measures: to indicate the relation between current food craving and negative affect, within-person correlation coefficients were calculated and included as ‘CravingNA_corr_’. The second EMA measure used the end-of-the day emotional eating review as ‘EndOfDayEE’.

To test our hypothesis of factorial validity, a measure model for EE_lat_ comprising DEBQ_emo_ + SEES_neg_ + CravingNA_corr_ + EndOfDayEE + DTE_hcal_ + Pleasantness_hcal_ was specified. Residual covariances between the same measure pair (i.e., two questionnaires, two EMA, and two laboratory measures) were specified to take method-specific variance into account [40]. In a second step, a model with correlations between EE_lat_ and health-relevant constructs (BMI, general eating disorder symptomatology measured with the EDE-Q8) to test concurrent validity, and restrained eating (measured with the restrained subscale of the DEBQ) to test construct validity, was specified. Confirmatory factor analysis was conducted in R version 4.1.2 [41] using the package lavaan version 0.6–10 [42] with robust maximum likelihood estimation method. For the CFA, fit indices were evaluated following established thresholds [CFI > 0.90, TLI > 0.90; [43], RMSEA < 0.08 [44], and SRMR close to 0.08 [45, 46]. Effects were considered as significant at *p* < 0.05. This study’s design and its analysis were not pre-registered, but the R-code and data sets for all analyzes can be accessed at https://osf.io/q8dtu/.

## Results

Descriptive statistics of measures and correlates of EE_lat_ are listed in Tables 2 and 3.

**Table 2:** Means (*M*) and standard deviations (SD) of measures and correlates of latent emotional eating

Variable	*M* (*SD*)
Dutch eating behavior questionnaire—emotional eating [1–5]	2.62 (0.72)
Dutch eating behavior questionnaire—restrained eating [1–5]	2.57 (0.76)
Salzburg emotional eating scale [1–5]	2.98 (0.51)
Eating disorder examination-questionnaire 8 [0–6]	2.11 (1.42)
Body mass index (in kg/m^2^)	25.9 (7.14)
Daily life: correlation craving–negative affect [− 1–1]	0.04 (0.18)
Daily life: end-of-day emotional eating [0–100]	51.7 (6.70)
Laboratory: desire to eat (difference score)	− 3.36 (21.2)
Laboratory: pleasantness (double difference score)	− 1.65 (16.7)

**Table 3:** Correlation matrix of measures submitted to the confirmatory factor analysis

Variable	DEBQ_emo_	DEBQ_res_	SEES_neg_	EDE-Q8	BMI	Craving-NA_corr_	EndOf-DayEE	DTE_hcal_	Pleasantness_hcal_
DEBQ_emo_	1								
DEBQ_res_	0.142	1							
SEES_neg_	0.491	0.049	1						
EDE-Q8	0.335	0.643	0.230	1					
BMI	0.343	0.292	0.352	0.514	1				
Craving-NA_corr_	0.215	− 0.091	0.174	− 0.006	0.044	1			
EndOf-DayEE	0.178	0.218	0.217	0.338	0.246	0.249	1		
DTE_hcal_	0.238	− 0.010	0.199	− 0.024	0.175	0.115	0.122	1	
Pleasant-ness_hcal_	0.259	− 0.033	0.117	0.071	0.127	0.117	0.136	0.581	1

*DEBQ*_*emo*_  Dutch eating behavior questionnaire—emotional eating subscale; *DEBQ*_*res*_ = Dutch eating behavior questionnaire—restrained eating subscale; *SEES*_*neg*_  Salzburg emotional eating scale—negative subscales; *EDE-Q8*  Eating disorder examination-questionnaire; *BMI*  Body mass index; *CravingNAcorr*  Correlation craving—negative affect; *EndOfDayEE*  End-of-day emotional eating; *DTE*_*hcal*_  desire to eat (high-calorie foods); *Pleasantness*_*hcal*_  pleasantness (high-calorie foods)

**Fig. 1:**
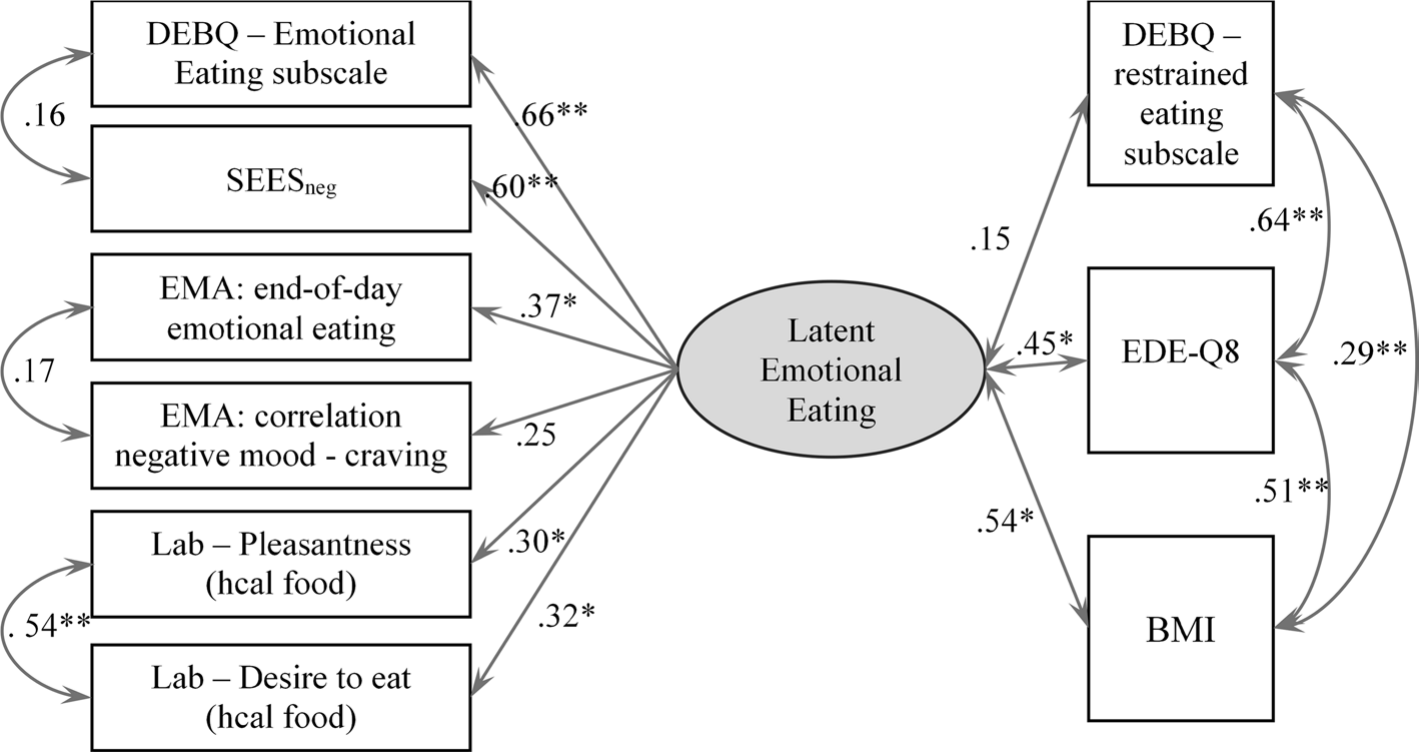
Standardized factor loadings of the confirmatory factor analysis linking each of the two measures of each of the three methods (questionnaires, EMA, laboratory; left side) to the latent emotional eating (EE_lat_) factor (middle). Relationships of EE_lat_ with variables of theoretical (restrained eating) and clinical (EDE-Q8, BMI) importance (right side). *hcal*  high calorie, *EMA*  ecological momentary assessment, *DEBQ*  Dutch Eating Behavior Questionnaire, *SEES*_*neg*_  Salzburg Emotional Eating Scale (subscales sadness, anger, anxiety), *BMI*  body mass index, *EDE-Q8*  Eating Disorder Examination Questionnaire, *p < 0.05; **p < 0.001

Indices indicated a good model fit for both the basic model (see Online Appendix 2) and the model with correlations (CFI = 0.97, TLI = 0.95, RMSEA = 0.05, and SRMR = 0.07). Results of the model with correlations are summarized in Fig. 1 and show support for a one-factorial EE_lat_ construct: all measures loaded on one construct at *p* < 0.05, with the exception of the current craving—negative affect correlation. This provides support for our first hypothesis. To account for the relatively small sample size, we also applied Bayesian statistic, which showed similar results (see Online Appendix 3).

With regard to our second hypothesis, the correlation between restrained eating and EE_lat_ was not significant; with a small effect size, thus no support for a cognitive theory of EE, but construct validity for our EE_lat_ was found. In contrast, higher BMI^1^ and higher eating disorder symptomatology correlated with higher EE_lat_, with medium to large effect sizes, supporting concurrent validity of EE_lat_.

## Discussion

EE is a popular concept in lay psychology and has clinical relevance, as it has been identified as a risk factor for (e.g., in children: [47])—and symptom of—disordered eating [6]. However, the EE literature is split on how to measure, explain, and interpret EE. This paper addressed measurement-related challenges (‘is there coherence across methods and measures?’) and the correlates of EE once measurement challenges are met (‘what are the correlates of a multi-measure EE?’). To do so, first, different measures of EE from laboratory, psychometric questionnaires, and EMA were obtained in the same individuals and then submitted to confirmatory factor analysis for a test of factorial validity. Second, restrained eating was measured to test construct validity and for a test of cognitive theories of EE. Finally, related clinical correlates of EE (BMI and general eating disorder symptomatology) were added to this model as a test of concurrent validity. Results support our factorial validity hypothesis: measures did indeed load on one latent construct, rather than measuring divergent constructs. Using the resulting latent ‘EE factor’ we then found it correlated with both BMI and general eating disorder symptomatology, but not with restrained eating.

### EE—elusive or evident? Theoretical implications

These findings have important implications. First, they partially contrast with some previous findings: some studies deemed EE to neither be detectable [16] nor measurable [17]. Others found support for EE, both in individuals with self-reported subclinical binge eating or restrained eating as well as a congruence between these self-reported traits and laboratory food intake [48]. Our results showed that both self-reported EE (in trait questionnaires as well as in daily states) and desire to eat after laboratory-induced negative emotions loaded on the same factor. This points to shared variance among those measures. Such shared variance can be seen as ‘agreement’ among measures about who is to be considered a high scorer on EE across measures. EMA measures were partially consistent with this: while intraday correlations between negative mood and food craving only showed a trend towards significance, end-of-day EE was clearly correlated with EE_lat_. This could be due to a difference between momentary and retrospectively reported EE that has been previously found in EMA studies [27, 49].

On a theoretical level, cognitive theories have deemed EE to be a form of unsuccessful restraint [16], i.e., EE being a temporal loss of control over food intake in response to a diet rule violation in restrained eaters. Hence, if restrained eating would underlie EE, we would have expected a significant positive correlation between restrained eating and our latent EE construct (EE_lat_). However, we found EE_lat_ unrelated to restrained eating. The results are in line with a recent review [50], which found the stress–eating relationship to be weaker in individuals with high restrained eating scores. Our results contrast with the meta-analyses by Cardi et al. [48] and Evers et al. [16] reporting emotion induced eating in restrained eaters. Yet, while Evers et al. [16] focused on laboratory studies of emotion-potentiated food intake, we included measures from three different methods and combined them through CFA and thus, used different data and a different statistical approach. Whereas the current study used food craving as a precursor for actual food intake, future research should also obtain objectively measured food intake in a study similar to ours to address this inconsistency.

In sum, although our results point to initial theoretical implications, a sophisticated differentiation or integration of different theories was beyond the scope of the present paper. A detailed examination of EE theories would also profit from including physiological measures (e.g., heart rate or cortisol levels) and further specifying of laboratory experiments, questionnaires and EMA studies to EE mechanisms according to different underlying theories.

### Clinical significance of EE

Second, this paper investigated constructs that have often been related to EE. This touches on issues of concurrent validity (what is EE related to?) and clinical relevance (can health topics be addressed or even predicted by EE?). We found concurrent validity for EE_lat_ through positive correlations with eating disorder symptomatology and BMI. This study’s sample was enriched with participants that have a higher BMI (see Table 1). In line with previous findings [7], our EE_lat_ construct was significantly related to higher BMI. This agrees with previous reports on the clinical significance of EE: in the light of a global obesity crisis, finding causes of overweight and obesity is crucial. A recent review found EE related to mood disorders and weight problems [51]. EE was further related to overeating, unhealthy eating, obesity, depressive symptoms, and distress. Another recent review found that interventions aiming at reducing EE also led to a significant weight reduction in individuals with overweight or [52]. Previous research also demonstrated the clinical importance of EE in eating disorders, such as AN, bulimia nervosa and binge-eating disorder [6, 7, 31]. Consistently across different countries, binge eating is more common in individuals with higher EE [53]. We recommend future research in samples including eating disorders to clarify clinical significance as we only measured sub-clinical variation in eating disorder symptoms here. Studies in emotionally disturbed individuals would speak to the specificity to the eating domain [51].

### Strength and limits

We are not aware of other studies employing a multi-method/multi-measure strategy to EE as applied here with three methods (questionnaire, laboratory and EMA) and two measures each. Applying such a strategy can result in a valid assessment of the EE construct as shown here. Clearly, this approach imposes high subject burden and high investment on the side of researchers and is thus not feasible in large scale studies. Yet, we believe that the CFA approach has various strengths that make it a promising tool for future theoretical and ‘proof of principle' research. The latent variable modelling approach separates construct variance (shared between the methods) from method variance, which is not possible with any of the single measures. We further ‘amplified’ this aspect by including two measures from each method. Thus, while each measure has its own shortcomings, these can be addressed in another measure. As indicated above, questionnaires suffer from biases (termed ‘triple recall bias’): participants have to remember eating occasions, emotional occasions and their causal relationship in the order emotions–eating (not the reverse). EMA measures, by contrast, limit these biases by asking momentary questions or questions relating to the past few hours. Both questionnaire and EMA methods, however, cannot ascertain causality. Here, emotion manipulation studies come into play, which, in turn have difficulties in naturalistic assessment of eating-related behaviors (which, again, are less of an issue in questionnaire and EMA studies). Thus, methods complement each other in compensating respective weaknesses. The covariance of these three methods thus are likely to result from ‘true’ covariance, since measurement error of each method domain should be uncorrelated (the degree of memory bias in questionnaire studies should not covary with the degree of eating behavior underreporting in the laboratory).

Yet, this paper provides no proof of the absolute effect of emotions on eating. That is, in contrast to the studies meta-analyzed in Evers et al. [16] we did not assess the increase in eating (or other eating behaviors) under negative emotions compared to a neutral state but only looked at the coherence of several measures of EE across individuals. We would expect that high scorers on our EE_lat_ factor should in fact show such emotion-related eating increase but testing this was beyond the scope of this paper. Likewise, it is unclear whether emotions cause an increase in eating across all individuals. As articulated elsewhere [54] we regard a ‘moderated EE concept’ with inter-individual differences as most likely: emotions can affect eating in some individuals and in those, direction (i.e., increase, decrease) might differ (i.e., emotion-related decrease in AN or appetite loss after severe stress). However, what follows from our concurrent validity findings is that individuals with a higher BMI and/or self-reported (subclinical) pathological eating show elevated EE_lat_. Causality, however, cannot be drawn from this correlation, i.e., EE might be a causative factor that explains the transition from an unhealthy eating trait to eating or weight pathologies or the reverse direction might be true: elevated eating and weight disorder symptoms changing emotional eating patterns. Longitudinal research manipulating EE_lat_, e.g., in treatment studies is needed here. In addition, our results only allow interpretations for ‘negative EE’, although also ‘positive EE’ has been proposed [17]. So-called ‘happy eating’ might have different underlying mechanisms, might be less related to psychopathology and, unlike negative EE, seems at an initial research stage. However, examining coherence between different methods with regard to positive EE might be worthwhile to obtain a bigger picture of general EE.

Generalizability of these findings is limited by the following factors: the female-only sample, the limited age range, an education level that might not be representable for the general population, the lack of actual food intake assessment, and the moderate sample size. Please note that because of this limited range in age and education, we refrained from controlling for these variables in the analyzes, as we do not think that these would change the results substantially. However, research suggests that EE differs according to gender [55], socioeconomical status [56] or age [57], so that enriching samples of future studies regarding these variables seems worthwhile. Regarding actual food intake, we argue that being monitored while eating in the context of a study creates an artificial eating situation and may thus not be more valid than our “proxies” of EE. While our two questionnaires measured differed in that regard (i.e., DEBQ assessing food craving, SEES_neg_ assessing eating behavior), both, the laboratory and the EMA measures did not assess actual food intake. Examining the translation of our food craving proxies into actual emotional eating behavior, as well as their coherence or divergence seems an interesting avenue for future research. Regarding the moderate sample size, we applied Bayesian statistics and could demonstrate similar findings with this analytic approach (see Online Appendix 3). It should be noted that given a sample size of *n* = 102, statistical power for testing a correlation coefficients equal *r* = 0.27 has an adequate power of 1—Beta = 0.80 at alpha = 0.05. Hence, there was not enough statistical power to test the empirically observed correlation between EE_lat_ and restrained eating subscale of the DEBQ with *r* = 0.14. Future studies could address these limitations by including a larger, mixed-sex sample.

### What is already known on this subject?

Emotional eating defines altered eating behavior in response to emotions and has been connected to disordered eating and weight disorders. Previous research showed mixed findings about the validity of the construct and is inconsistent with regard to how to best measure it. Methodologically strong studies are needed to shed light on the measurability of emotional eating and its relation to other constructs, i.e., restrained eating, BMI, and other indicators for pathological eating.

### What this study adds?

The present study showed that the latent construct of EE can validly be assessed with a multi-method/multi-measure approach including psychometric, experimental and naturalistic data, using structural equation modeling. Moreover, this latent EE construct positively relates to higher BMI and greater eating disorder symptomatology but not to restrained eating, supporting concurrent and construct validity. Hence, the study contributes to the ongoing debate about the validity of EE by applying a new analytical approach (e.g., [58, 59]).

### Supplementary Information

Below is the link to the electronic supplementary material.

Supplementary file1 (DOCX 126 KB)

Supplementary file2 (DOCX 35 KB)

Supplementary file3 (DOCX 35 KB)

Supplementary file4 (DOCX 236 KB)

## Data Availability

All data, analysis code, and research materials are available at https://osf.io/q8dtu/.

## Footnotes

1 Online Appendix 4 contains the model with subjective BMI data only. Significance and direction of results did not change substantially.
