# Supplementary material for: Emotional eating: elusive or evident? Integrating laboratory, psychometric and daily life measures
Source: Eat Weight Disord. 2023 Sep 13;28(1):74. doi: 10.1007/s40519-023-01606-8 (PMC10499733; doi:10.1007/s40519-023-01606-8)
Supplement: Supplementary file 1 — Supplementary file1 (DOCX 126 KB) [file 40519_2023_1606_MOESM1_ESM.docx]

Appendix 1 – Food Pictures

| Food Picture | Food Picture Number | Food Picture | Food Picture Number |
| --- | --- | --- | --- |
| 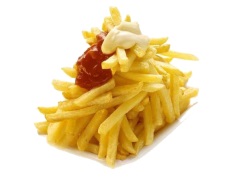 | 0022 | 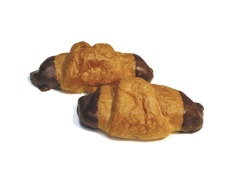 | 0184 |
| 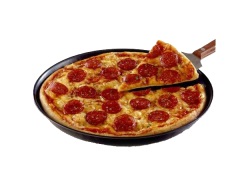 | 0061 | 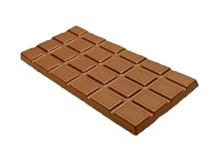 | 0286 |
| 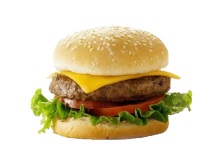 | 0065 | 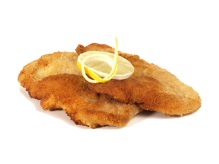 | 0309 |
| 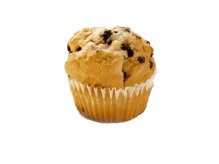 | 0080 | 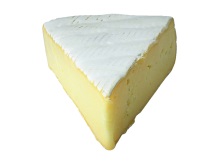 | 0519 |
| 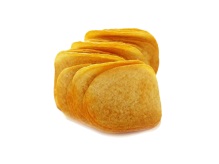 | 0117 | 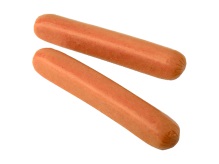 | 0535 |
| 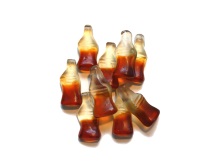 | 0153 | 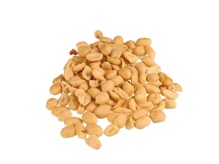 | 0183 |
| 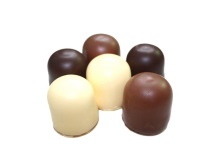 | 0161 |  |  |

*Figure A1*. Thirteen high-caloric foods that were used in the laboratory measures of emotional food cue reactivity, derived from the foodpics_dataset extended [1].

1. Blechert, J., et al., *Food-Pics_Extended—An Image Database for Experimental Research on Eating and Appetite: Additional Images, Normative Ratings and an Updated Review.* Frontiers in Psychology, 2019. **10**.
