# Supplementary material for: Emotional eating: elusive or evident? Integrating laboratory, psychometric and daily life measures
Source: Eat Weight Disord. 2023 Sep 13;28(1):74. doi: 10.1007/s40519-023-01606-8 (PMC10499733; doi:10.1007/s40519-023-01606-8)
Supplement: Supplementary file 2 — Supplementary file2 (DOCX 35 KB) [file 40519_2023_1606_MOESM2_ESM.docx]

Appendix 2 – Basic Model

SEES_neg_

DEBQ – Emotional Eating subscale

EMA: end-of-day emotional eating

EMA: correlation negative mood - craving

Lab – Pleasantness (hcal food)

Lab – Desire to eat (hcal food)

.25

.16

. 52*

.63*

.51*

.33*

.33

.36*

.38*

*Figure A2.* Standardized factor loadings of the confirmatory factor analysis and correlations between measures of the same method. Fit indices for the model were CFI = 1, TLI = 1.11, RMSEA = 0, and SRMR = .021. Note: hcal = high calorie; EMA = ecological momentary assessment; DEBQ = Dutch Eating Behavior Questionnaire; SEES_neg_ = Salzburg Emotional Eating Scale (subscales sadness, anger, anxiety); *** = *p* < .05.

Für submission raus

> model_basic <- '

+ # measurement model

+ EElat =~ DEBQ_Emotionalitaet_gm + SEES_na_gm + DTE_hcal_gm + PLEA_hcal_gm + COR_EMA_z + eatmood_gm

+ #residual correlations

+ COR_EMA_z ~~ eatmood_gm

+ DTE_hcal_gm ~~ PLEA_hcal_gm

+ DEBQ_Emotionalitaet_gm ~~ SEES_na_gm

+ '

> fitbasic <- cfa(model = model_basic, data = SEM3, std.lv=T, estimator="MLR")

Estimator ML

Optimization method NLMINB

Number of model parameters 15

Used Total

Number of observations 102 103

Model Test User Model:

Standard Robust

Test Statistic 3.347 3.138

Degrees of freedom 6 6

P-value (Chi-square) 0.764 0.791

Scaling correction factor 1.067

Yuan-Bentler correction (Mplus variant)

Model Test Baseline Model:

Test statistic 98.315 94.976

Degrees of freedom 15 15

P-value 0.000 0.000

Scaling correction factor 1.035

User Model versus Baseline Model:

Comparative Fit Index (CFI) 1.000 1.000

Tucker-Lewis Index (TLI) 1.080 1.089

Robust Comparative Fit Index (CFI) 1.000

Robust Tucker-Lewis Index (TLI) 1.092

Loglikelihood and Information Criteria:

Loglikelihood user model (H0) -1328.995 -1328.995

Scaling correction factor 1.023

for the MLR correction

Loglikelihood unrestricted model (H1) -1327.322 -1327.322

Scaling correction factor 1.035

for the MLR correction

Akaike (AIC) 2687.991 2687.991

Bayesian (BIC) 2727.365 2727.365

Sample-size adjusted Bayesian (BIC) 2679.986 2679.986

Root Mean Square Error of Approximation:

RMSEA 0.000 0.000

90 Percent confidence interval - lower 0.000 0.000

90 Percent confidence interval - upper 0.089 0.080

P-value RMSEA <= 0.05 0.854 0.883

Robust RMSEA 0.000

90 Percent confidence interval - lower 0.000

90 Percent confidence interval - upper 0.087

Standardized Root Mean Square Residual:

SRMR 0.025 0.025

Parameter Estimates:

Standard errors Sandwich

Information bread Observed

Observed information based on Hessian

Latent Variables:

Estimate Std.Err z-value P(>|z|) Std.lv Std.all

EElat =~

DEBQ_Emtnltt_g 0.498 0.165 3.016 0.003 0.498 0.697

SEES_na_gm 0.270 0.124 2.174 0.030 0.270 0.534

DTE_hcal_gm 7.614 3.320 2.294 0.022 7.614 0.373

PLEA_hcal_gm 6.327 3.172 1.995 0.046 6.327 0.397

COR_EMA_z 0.055 0.026 2.107 0.035 0.055 0.298

eatmood_gm 2.047 1.078 1.899 0.058 2.047 0.307

Covariances:

Estimate Std.Err z-value P(>|z|) Std.lv Std.all

.COR_EMA_z ~~

.eatmood_gm 0.196 0.121 1.624 0.104 0.196 0.174

.DTE_hcal_gm ~~

.PLEA_hcal_gm 137.902 45.314 3.043 0.002 137.902 0.498

.DEBQ_Emotionalitaet_gm ~~

.SEES_na_gm 0.043 0.092 0.465 0.642 0.043 0.196
