# Supplementary material for: Emotional eating: elusive or evident? Integrating laboratory, psychometric and daily life measures
Source: Eat Weight Disord. 2023 Sep 13;28(1):74. doi: 10.1007/s40519-023-01606-8 (PMC10499733; doi:10.1007/s40519-023-01606-8)
Supplement: Supplementary file 3 — Supplementary file3 (DOCX 35 KB) [file 40519_2023_1606_MOESM3_ESM.docx]

Appendix 3 – Bayes Analyses

SEES_neg_

DEBQ – Emotional Eating subscale

EMA: end-of-day emotional eating

EMA: correlation negative mood - craving

Lab – Pleasantness (hcal food)

Lab – Desire to eat (hcal food)

DEBQ – restrained eating subscale

BMI

EDE-Q8

.27

.63

.49

.18

.17

. 50

.65

.59

.37

.25

.31

.33

.12

.40

.49

*Figure A3.* Standardized factor loadings obtained from Bayes analyses of the confirmatory factor analysis linking each of the two measures of each of the three methods (questionnaires, EMA, laboratory; left side) to the latent emotional eating (EE_lat_) factor (middle). Relationships of EE_lat_ with variables of theoretical (restrained eating) and clinical (EDEQ, BMI) importance (right side). Note: hcal = high calorie; EMA = ecological momentary assessment; DEBQ = Dutch Eating Behavior Questionnaire; SEES_neg_ = Salzburg Emotional Eating Scale (subscales sadness, anger, anxiety); BMI = body mass index; EDE-Q8 = Eating Disorder Examination Questionnaire.
