# Supplementary material for: Emotional eating: elusive or evident? Integrating laboratory, psychometric and daily life measures
Source: Eat Weight Disord. 2023 Sep 13;28(1):74. doi: 10.1007/s40519-023-01606-8 (PMC10499733; doi:10.1007/s40519-023-01606-8)
Supplement: Supplementary file 4 — Supplementary file4 (DOCX 236 KB) [file 40519_2023_1606_MOESM4_ESM.docx]

Appendix 4 – Model with Subjective BMI Values

*Figure A4.* Standardized factor loadings of the confirmatory factor analysis linking each of the two measures of each of the three methods (questionnaires, EMA, laboratory; left side) to the latent emotional eating (EE_lat_) factor (middle). Relationships of EE_lat_ with variables of theoretical (restrained eating) and clinical (EDEQ, BMI_sub_) importance (right side). Note: hcal = high calorie; EMA = ecological momentary assessment; DEBQ = Dutch Eating Behavior Questionnaire; SEES_neg_ = Salzburg Emotional Eating Scale (subscales sadness, anger, anxiety); BMI_sub_ = body mass index based on subjective weight; EDE-Q8 = Eating Disorder Examination Questionnaire; *** = *p* < .05; ** = *p* < .001
